# Correlation of Endometrial Glycodelin Expression and Pregnancy Outcome in Cases with Polycystic Ovary Syndrome Treated with Clomiphene Citrate Plus Metformin: A Controlled Study

**DOI:** 10.1155/2015/278591

**Published:** 2015-02-28

**Authors:** Selda Uysal, Ahmet Zeki Isik, Serenat Eris, Seyran Yigit, Yakup Yalcin, Pelin Ozun Ozbay

**Affiliations:** ^1^Gynecology and Obstetrics Department, Ataturk Training and Research Hospital, Basin Sitesi, Yesilyurt, 35360 İzmir, Turkey; ^2^Gynecology and Obstetrics Department, Izmir University Hospital, 35360 İzmir, Turkey; ^3^Gynecology and Obstetrics Department, Isparta Obstetrics and Pediatrics Hospital, 32000 Isparta, Turkey; ^4^Pathology Department, Ataturk Training and Research Hospital, Basin Sitesi, Yesilyurt, 35360 İzmir, Turkey; ^5^Gynecologic Oncology Department, Suleyman Demirel University Hospital, 32000 Isparta, Turkey; ^6^Gynecology and Obstetrics Department, Aydın Obstetrics and Pediatrics Hospital, 09100 Aydın, Turkey

## Abstract

*Objective*. The purpose of this study was to evaluate the relationship between clomiphene citrate (CC) plus metformin treatment and endometrial glycodelin expression and to then correlate this relationship with pregnancy outcomes. *Material and Methods*. A total of 30 patients diagnosed with polycystic ovary syndrome (PCOS) according to the Rotterdam criteria constituted our study group. All had been admitted to the gynecology outpatient clinic between June 1, 2011, and January 1, 2012, for infertility treatment. Our control group consisted of 20 patients admitted for routine Pap smear control. They had no history of infertility and were not using contraceptives and they were actively attempting pregnancy. Midluteal progesterone measurement and pipelle endometrial biopsies were performed with both groups. For PCOS patients, metformin treatment was initiated right after the biopsy and CC was added in the second menstrual cycle. Pipelle endometrial biopsies were repeated. Histological dating and immunohistochemistry for glycodelin were performed by a single pathologist who was blinded to the patients' clinical data. *Result(s)*. The posttreatment ovulation rate in the study group was 93.3%. No pregnancies were achieved in either group when glycodelin expression was not present, even in the presence of ovulation. When glycodelin expression was high in PCOS group, the pregnancy rate was 60% and all pregnancies ended in live births. In weak expression group, however, three out of four pregnancies ended as early pregnancy losses. *Conclusion(s)*. Endometrial glycodelin expression is an important predictor of pregnancy outcomes in both PCOS and fertile groups.

## 1. Introduction

Polycystic ovary syndrome (PCOS) is the most common cause of anovulatory infertility and occurs at a rate between 6% and 12% during the reproductive period [1–3]. This endocrinopathy, the etiology of which has not been fully elucidated, begins in the postmenarcheal period and continues until the premenopausal period [3, 4]. It is characterized by chronic anovulation and hyperandrogenemia [1–4]. In addition to anovulation-related infertility, the frequency of early pregnancy loss in PCOS is three times higher than in the normal population [2, 3]. In cases with PCOS, the rate of pregnancy loss following the clinical identification of assisted or spontaneous pregnancy is 30%–50% [1–3].

The most important feature of PCOS is compensatory hyperinsulinemic insulin resistance [1–5]. By stimulating ovarian androgen production and decreasing serum sex hormone-binding globulin (SHBG) concentrations, hyperinsulinemic insulin resistance leads to hyperandrogenism [1, 2] and anovulation [1, 2, 6–8]. With the suppression of endometrial glycodelin levels, endometrial functions are impaired, the preimplantation environment is adversely affected, and the rate of early pregnancy loss increases [1, 2].

Glycodelin is a major glycoprotein induced by progesterone and secreted from the secretory and decidual endometrium during the luteal phase [1, 2, 9, 10]. It is observed during the ovulatory cycles and facilitates implantation by inhibiting the immune response [9, 10]. The downregulation of maternal immune response against fetal alloantigenicity is achieved via the T-cells [9–11]. Glycodelin induces the apoptosis of monocytes before their differentiation into macrophages through the mitochondrial pathway, without affecting phagocytic activity. The decrease of glycodelin in the endometrium and serum is associated with growth retardation of endometrium, early pregnancy loss, and recurrent early pregnancy loss [1, 2].

Insulin-sensitizing drugs and metformin in particular not only are involved in the correction of insulin resistance but also serve to treat reproductive abnormalities [3, 4]. Biguanide metformin is the most frequently used insulin-sensitizing agent in type 2 diabetes mellitus (DM). Aside from the liver, biguanide metformin also has an effect on the skeletal muscles, the adipose tissue, the endothelium, and the ovaries. It increases ovulation, corrects the menstrual cycle, and decreases the levels of serum androgens. It achieves its reproductive effects both via insulin and through direct effects on ovarian functions. It is an effective agent for ensuring ovulation [1–4].

In PCOS, hyperinsulinemic insulin resistance decreases the concentration of circulating glycodelin [1, 2]. In nonpregnant PCOS patients, metformin increases serum glycodelin levels threefold during the luteal phase [1, 2].

The aim of our study was to demonstrate the effect of clomiphene citrate (CC) plus metformin treatment on endometrial glycodelin expression in PCOS patients and then correlate the relationship with rates of successful pregnancies and pregnancy outcomes. The effect of the intensity of endometrial glycodelin expression on pregnancy outcome was also controlled with the fertile group of women.

## 2. Materials and Methods

A total of 30 patients diagnosed with PCOS according to the Rotterdam criteria [12] constituted our study group. All were admitted to the gynecology outpatient clinic for fertility treatment between June 1, 2011, and January 1, 2012. Our control group consisted of 20 patients admitted for routine Pap smears. These women had no history of infertility and no contraceptive use and they were actively attempting pregnancy. Nine out of these twenty patients had previously given live births (45%). Before entering this prospective cohort study, the protocol was explained to patients and a written consent was obtained from patients agreeing to participate. Consent forms and the protocol were approved by the local ethical committees. The physical and pelvic examination findings of these healthy patients, as well as their menstrual cycles, were normal, and none of them had a history of hormonal drug or intrauterine device (IUD) use in the previous three months. The study and control groups consisted entirely of nonsmoking patients between 20 and 40 years of age with no history of systemic or clinically active diseases. All women were naïve to clomiphene and had not undergone any significant treatment for infertility or ovulation induction earlier. Patients with hyperprolactinemia, thyroid disorder, male factor, suspected tubal factor, or endometriosis were not included in the study. There were no weight or Body Mass Index (BMI) restrictions. The complete blood-count values, follicle stimulating hormone (FSH), luteinizing hormone (LH), estradiol (E2), prolactin, thyroid stimulating hormone (TSH), total and free test, dehydroepiandrosterone sulfate (DHEA-S), 17-hydroxyprogesterone (17-OHP), luteal phase progesterone, and oral glucose tolerance test (OGTT) levels were measured during the routine clinical chemistry tests of all patients on the third day of the cycle. The BMI indices of the patients were also measured.

Following the completion of their routine tests, patients diagnosed with PCOS were administered with medroxyprogesterone acetate (MPA) 5 mg b.i.d. for six days. To perform endometrial dating, the level of progesterone was evaluated and a pipelle biopsy was taken in the period between the 21st and 24th days following the patients' menstrual bleeding. Tissue glycodelin levels were assessed in these endometrial samples.

Following biopsy, 16 patients with BMI <25 were administered metformin 500 mg b.i.d., while 14 patients with BMI >25 were given a dosage of 500 mg t.i.d. Blood biochemistry samples were obtained again following the spontaneous or induced second menstrual bleeding. Between the 5th and 9th days of the second menstrual cycle, ovulation induction was performed with clomiphene citrate under ultrasound monitoring. When the dimensions of the leading follicle reached between 18 mm and 22 mm, hCG was administered (Ovitrelle 250 *μ*g, Merck Serono, Istanbul, Turkey). The serum progesterone level and ovulation were evaluated 9–11 days later. Pipelle biopsies were taken from 21–24 days after the beginning of the menstrual cycle. Tissue glycodelin levels were also assessed in these endometrial samples in addition to histological endometrial dating. For the study group, both pre- and posttreatment biopsies were conducted. We did not give any treatment to the control group and only one biopsy was taken of the fertile patients. All patients were followed up for three cycles. Any dose changes were made during the follow-up visits. Metformin treatment was discontinued after fetal cardiac activity observed.

### 2.1. Evaluation of Endometrial Biopsies

A pathologist evaluated all biopsies and confirmed that the specimens had normal endometria with no signs of pathology. Endometrial dating was performed as described in the literature [13]. Immunohistochemical analyses for glycodelin were carried out by using the horseradish peroxidase labelled streptavidin-biotin (HRP-LSAB) method. Briefly, 5 *μ*m thick sections were cut from each paraffin block, deparaffinized, rehydrated, and subjected pre-treatment to enhance immunoreactivity, for which ethylenediaminetetraacetic acid (EDTA) 1/150 dilution was used for 25 minutes in a pretreatment procedure. Placental protein 14/glycodelin A antibody [EP870Y] (ab53289 Abcam, Cambridge, MA, USA) was used as a primary antibody 1 : 150 dilution for one hour at room temperature. The second antibody used was Horse Radish Peroxidase (DAKO, Glostrup, Denmark). Diaminobenzidine (DAB) was used as a chromogen for reaction visualization. Finally, the sections were counterstained with Mayer's hematoxylin, then dehydrated, cleared with xylene, and mounted with coverslips using a permanent mounting medium.

**Figure 1:**
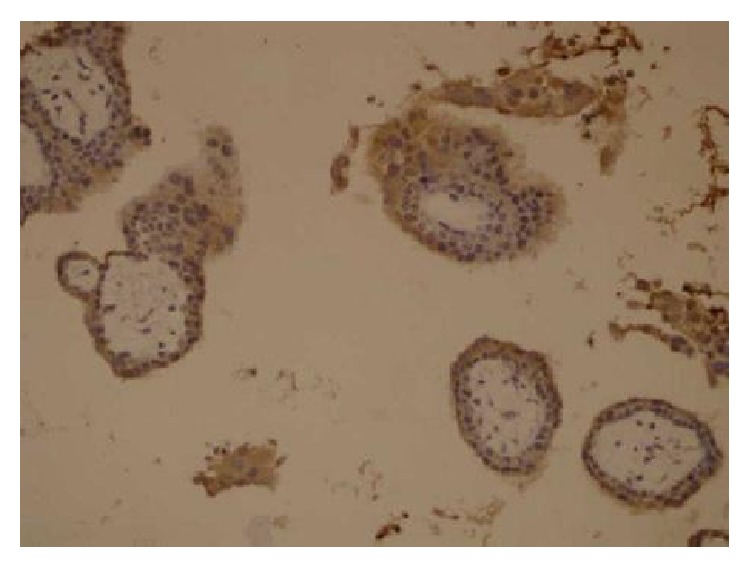
Immunohistochemical staining of glycodelin positive control in chorion villous tissue (×20).

Cytoplasmic staining of trophoblastic cells in chorionic villous tissue was used as a positive control (Figure 1).

The antibody reacts with secretory glands and the luminal surface of normal secretory endometrium from the fifth postovulatory day. In normal proliferative endometrium, the antibody does not react with the antibody. We found no stromal staining.

**Figure 2:**
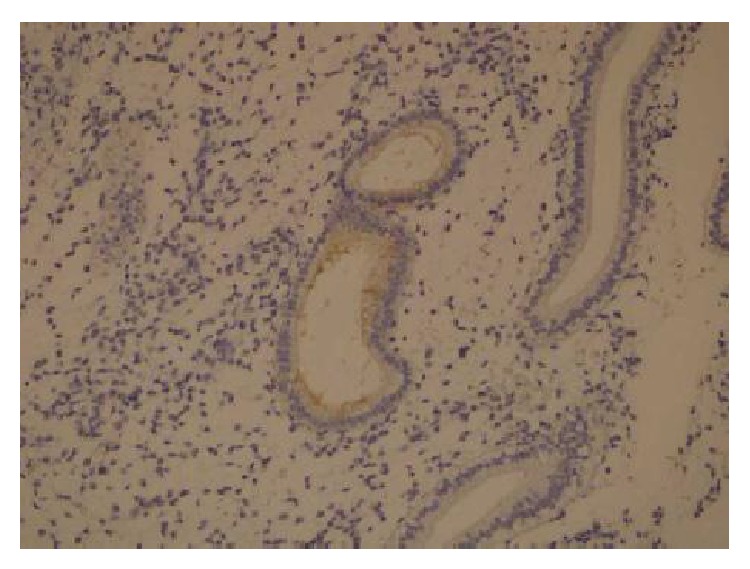
Weak glycodelin expression (×20).

**Figure 3:**
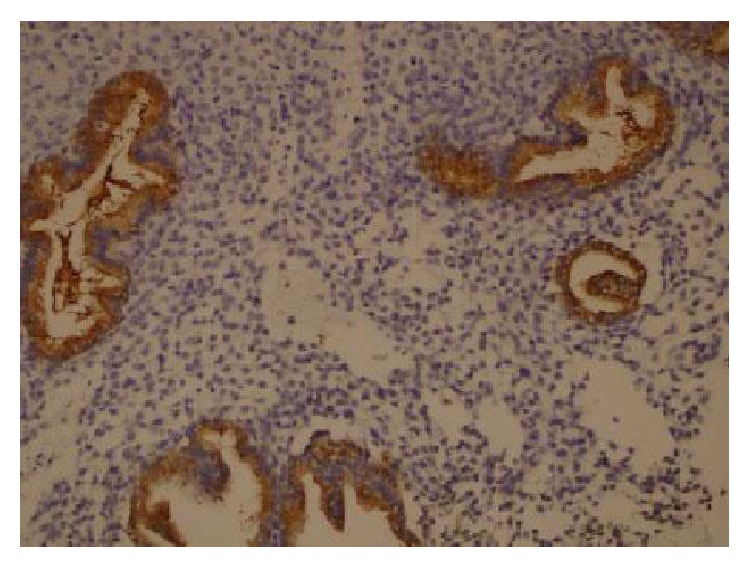
Strong glycodelin expression (×20).

For immunohistochemical analysis, all endometrial tissues were examined at low magnification (×10). For the antibody in the glandular epithelial cells, at ×20 magnification, the percentage of stained cells was determined semiquantitatively and subjectively. The immunostaining was evaluated on a scale of 0–3 points according to the percentage of cytoplasmic glandular cells that stained positively. Less than 5% of glandular cell staining was termed negative, 6–25% positivity was rated as 1 point, 26–50% positivity was rated as 2 points, and more than 50% positivity was rated as 3 points. According to the staining intensity, the immunostaining was evaluated on a scale of 1–3 points (1 point: weak; 2 points: moderate; 3 points: strongly positive). Points for the intensity staining (IS) and percentage (%S) of positive cells were added together, and an overall score (OS 0–2) was assigned. Staining was categorized into three groups based on the OS as follows: 1 point was negative expression (0 or <5% cells stained regardless of intensity); 2 points were a weak expression (OS, 1); 2 to 3 points; 3 points were a strong expression (OS, 2); 4 to 6 points; (Figures 2-3).

### 2.2. Statistical Analysis

Statistical analysis of the collected data was performed by using SPSS 16.0 (SPSS, Inc., Chicago, IL, USA). Fisher's Exact Test and Pearson's chi square test were used for the statistical comparisons for categorical data between the groups. The Kruskal-Wallis* H* test was used for the statistical comparisons for continuous data between more than two groups and the Mann Whitney *U* test was used for the statistical comparisons for continuous data between the two groups. *P* values of <0.05 or less were considered as statistically significant.

For patients who became pregnant following the conduction of the study, the cycle in which they become pregnant, the outcome of the pregnancy, and complications were all recorded and evaluated.

## 3. Results

The mean age of the 30 patients in the study group was 25.6 ± 3.78, while the mean age of the 20 patients in the control group was 26.11 ± 4.21. The mean BMI values were determined to be 26.24 ± 4.91 for the study group and 24.51 ± 3.21 for the control group. The BMI values of the study group were significantly higher than the values for the control group (*P* < 0.05). When the laboratory values were evaluated, the pretreatment (predrug) LH and free testosterone values were significantly higher than the values for the control group, while the FSH and pretreatment progesterone values were significantly lower (*P* < 0.05). No significant differences were identified between the groups with respect to the other laboratory variables (Table 1). Posttreatment midluteal progesterone levels increased and a decrease was identified in the free testosterone values.

Among patients in the study group, a significant difference was identified between the pre- and posttreatment endometrial biopsy results (*P* < 0.05). There were 26 (86.7%) proliferative and 4 postovulatory (13.3%) endometria in the pretreatment period among PCOS patients (i.e., the study group); the rate of ovulation among these 30 patients reached 93.3% (28/30) following treatment. In the control group, the endometrial biopsy results in 19 out of 20 cases (95%) were postovulatory.

While no glycodelin expression was identified during the pretreatment period in the biopsy specimens of the 30 patients belonging to the study group, including the ovulatory patients, glycodelin was identified in 22 of the 28 patients (73.3%) who were classified as ovulatory during the posttreatment period. A statistically significant difference was identified between the pre- and posttreatment glycodelin levels of the study group (*P* < 0.05).

In the control group, no glycodelin was identified in 3 patients (15%) whereas glycodelin was positive in 17 patients (85%).

Spontaneous pregnancies occurred in 12 patients (60%) within the control group. All twelve were in the glycodelin positive group (12/17).

With respect to glycodelin positivity, pregnancies occurred in three out of five patients who showed weak staining positivity (60%). Nine out of 12 patients who had shown strong staining positivity (75%) also became pregnant.

In brief, 10 of 30 patients (33.3%) within the study group became pregnant. For both the study and control groups, a total of 22 patients who displayed glycodelin staining positivity became pregnant. In this group, the pregnancy rate was 45.4%. With respect to glycodelin positivity, four of the pregnancies occurred among women with positivity values of 1, while six pregnancies resulted after positivity values of 2.

Within the study and control groups, the increase in pregnancy rates following an increase in glycodelin positivity was found to be statistically significant *P* value for study and control groups were 0.027 and 0.030, respectively (Fisher's Exact Test, *P* < 0.05) (Table 2).

While two out of total twelve pregnancies in the control group resulted in miscarriages, ten of the pregnancies resulted in live births. With regard to the pregnancy cycles, six pregnancies occurred during the first cycle, while six other pregnancies occurred during the second and third cycles (the patients were followed for a total of three cycles).

In the study group, seven out of ten pregnancies occurred in the first cycle, while the remaining three pregnancies occurred in the second and third cycles. In addition, while three out of ten pregnancies resulted in early pregnancy loss (EPL), seven of the pregnancies resulted in live births.

When the glycodelin positivity rates were evaluated, one of the two pregnancy losses in the control group displayed glycodelin (+) positivity, while the other had glycodelin (++) positivity. Within the study group, one of four pregnancies with glycodelin (+) positivity progressed successfully, while three resulted in early pregnancy loss. The early pregnancy loss rate was 75% in the glycodelin (+) study group.

Five out of seven of the pregnancies among the study group resulted in live and healthy births. In two of our cases, a caesarean section was performed at the 36th week due to placental abruption. No maternal and neonatal complications were observed.

When the mean progesterone values of the study group were evaluated for glycodelin positivity, no significant difference was identified between the groups (Table 3).

## 4. Discussion

Implantation involves the adhesion of the embryo to the decidua, its descent towards the basal membrane, and its invasion into the stroma. This process between the endometrium and the embryo is regulated by a complex system that involves the interaction of growth factors, hormones, adhesion molecules, extracellular matrices, and prostaglandins. Although assisted reproductive techniques are able to achieve fertilization rates in 70%–80% of cases, the average rate of resulting live births remains at only 35%–40%. This observation can be explained by implantation failures and deficiencies in endometrial receptivity [9].

Glycodelin is an important and promising indicator of implantation. It is involved in the processes pertaining to endometrial receptivity and implantation [9]. Low levels of glycodelin during the midcycle fertile window are conducive to fertilization, while higher glycodelin levels during the midluteal implantation window (between day 19 and day 24 during the 28-day cycle) allow for implantation to take place [14].

It has been demonstrated that the expression of glycodelin increases in the endometrium during the implantation window and late luteal phase, starting from postovulatory day five [14, 15]. Glycodelin has apoptotic [16] and antiproliferative [17] effects on T-lymphocytes, as well as immunosuppressive and inhibiting effects on natural killer (NK) lymphocytes. It ensures implantation by protecting the embryo from the maternal immune system [3, 9]. In other words, it plays a significant role in the early interactions between the embryo and the endometrium [9].

In anovulatory cycles as well as cases with PCOS, low levels of serum and endometrial glycodelin are observed; as a result, endometrial functions are impaired and the preimplantation environment is rendered less suitable [18]. It is known that compensatory hyperinsulinemic insulin resistance has the same effect in cases with PCOS [1, 2, 19]. Thus, low levels of glycodelin can lead to implantation failures, early pregnancy losses, and recurrent pregnancy losses.

In hyperinsulinemia, signals directly and indirectly originating from the endometrial stroma are inhibited, thus contributing to low glycodelin concentrations [1, 2]. Metformin treatment is known to decrease the levels of serum insulin, glucose, and androgen while increasing serum SHBG [1, 6–8]. In another study, the concentrations of circulating glycodelin were found to have increased nearly threefold during the CC-induced postovulatory luteal phase as a result of metformin use [1].

In their study, Li et al. [20] evaluated the levels of glycodelin in the endometrial flushings of fertile subjects with normal cycles. Glycodelin levels were low during the proliferative phase and the periovulatory fertile period. However, in the early luteal phase, levels increased starting from the sixth day after the LH peak and reached their maximum during the late luteal phase. Similarly, Mylonas et al. [10] found levels increasing significantly in endometrial tissue samples during the late luteal phase. Kao et al. [21] on the other hand evaluated the genes regulating glycodelin in endometrial tissue. Glycodelin genes expressions during the secretory phase were found to be 14.6 times higher in comparison to the proliferation phase. Brown et al. found that glycodelin expression began in the endometrial glands on the 16th day of the cycle in both controlled ovarian hyperstimulation and natural cycles and increased progressively over time [22].

The goal of our study was to ensure ovulation with CC+ metformin treatment, to increase glycodelin secretion by reducing serum insulin levels with metformin treatment, and to identify the effects of this treatment on luteal phase endometrial functions and the preimplantation environment in patients with PCOS.

In our study, the pretreatment ovulation rate of the 30 patients with PCOS was 13.3%, and no glycodelin was found in their endometrial samples, including those who were identified as being ovulatory. Following CC+ metformin treatment, 28 of them (93.3%) ovulated and a glycodelin expression rate of 73.3% was obtained for 22 of the 30.

No pregnancies occurred among women in either group when glycodelin expression did not occur during ovulation. In the study group, where all patients had PCOS, the pregnancy rate was 60% and all pregnancies resulted in live births when glycodelin expression had been strong. However, among the low-expression group, three out of four pregnancies ended in early miscarriages.

Serum glycodelin concentrations are found to be low during early and recurrent pregnancy losses [1, 23]. This decrease occurs especially in the endometrium [1, 24]. Glycodelin A is secreted from the decidual endometrium and is found in the amniotic fluid. Glycodelin A protects the embryo from the maternal immune system during implantation. It is also observed in the endometrium during the entrance of the embryo into the uterine cavity [1]. Deficiencies in endometrial glycodelin production can lead to local immunological responses. Observational studies have demonstrated that insulin resistance adversely affects endometrial functions, reduces the amount of circulating glycodelin, and increases the risk of pregnancy loss [2, 23]. In comparison to normal pregnancies, a significant decrease is observed in the serum glycodelin levels of pregnant women with PCOS during the first trimester, especially during the first two months. These levels return to normal between the 9th and 11th weeks. This decrease and fluctuation in glycodelin levels are significant with regard to the early and recurrent pregnancy losses observed in PCOS. Impaired glycodelin secretion limits the ability to control the maternal immune response against the embryo; at the same time, deficiencies related to the decrease in glycodelin concentrations arise in the implantation environment [4, 14, 25].

The rate of early pregnancy loss (EPL) is three times higher in PCOS patients [2, 26, 27]. While the rate of EPL is 10%–15% within the normal population [2, 28], it is 30%–50% among women with PCOS [2].

Insulin-sensitizing agents such as metformin decrease EPL in patients with PCOS by increasing the levels of glycodelin [2, 6].

In the study of Salim et al. [28], 20 patients with recurrent EPL and 16 fertile controls were evaluated. For the EPL group, a significant decrease was identified in the glycodelin levels of the uterine flushings, which were performed on the 7th day following the LH peak.

Dalton et al. [29] compared 49 patients with recurrent EPL and 15 fertile women. In that study, glycodelin levels in the serum and uterine flushings were assessed between the 10th and 12th days of the LH peak. While no differences were identified in the serum glycodelin levels, the uterine flushing levels were very low in the recurrent EPL group. In the ensuing stages of the study, the uterine flushing glycodelin levels of cases that resulted in abortus were lower in comparison to cases resulting in live births, while no difference was identified in the serum values.

Glycodelin was also assessed in ART cycles. Westergaard et al. [30] assessed serum glycodelin levels in the pretreatment cycle and the treatment cycle in 20 patients with planned ICSI on the 2nd, 8th, 12th, 14th, 20th, 24th, and 28th days of their cycles. In comparison to the other thirteen patients who are unable to conceive, a significantly lower level of glycodelin was identified in the seven patients who became pregnant on the treatment and two pretreatment cycles while a significantly higher level of glycodelin was identified during the late luteal phase of the cycle in which they became pregnant.

Chryssikopoulos et al. [31] and Liu et al. [32] identified higher serum glycodelin values on the oocyte retrieval and embryo transfer days of the women that became pregnant.

Although glycodelin is mainly induced by progesterone, the timing of its expression is not compatible or synchronous with progesterone levels [30]. In other words, expression of glycodelin is relatively delayed from the initiation of progesterone release [14, 20].

When we evaluated the mean progesterone values of our patients according to glycodelin positivity, no significant difference was identified between the two groups. This demonstrates that progesterone values cannot be used as a clear indicator of endometrial receptivity and implantation.

One of the significant advantages of our study was the ability to directly observe glycodelin levels in the endometrium. When the serum values were evaluated, we found that glycodelin was also produced in the extrauterine tissues (i.e., bone marrow, sweat glands, breasts, and ovaries). Although insulin primarily affects endometrial glycodelin secretion, it may also affect expression in these tissues.

Our study has demonstrated that, depending on its level of expression, glycodelin is associated with pregnancy outcome. In cases with PCOS, it was observed that glycodelin expression levels, rather than progesterone, were important during the administration of CC and metformin combination. Those patients with poor or little glycodelin expression had difficulties in achieving pregnancies or experienced EPLs. This information may be helpful for patients with early pregnancy losses or those who experience recurrent implantation failure in assisted reproductive techniques. In this group of patients, new studies could be planned and performed to assess whether metformin increases expression in patients with a low level of midluteal glycodelin expression. Further studies pertaining to this subject obviously are necessary.

In conclusion, according to both the results of our study and recent literatures [1, 2, 30–32], it has been demonstrated that insulin resistance and hyperinsulinemia lead to delays in the endometrial function of women with PCOS, yet they decrease the concentration of circulating glycodelin. They also adversely affect the implantation environment and lead to infertility and early and recurrent pregnancy losses. Metformin, on the other hand, serves to increase the levels of glycodelin.

## Figures and Tables

**Table 1:** Mean distribution of age, BMI, and laboratory values in the study and control groups.

	Study group		Control group		*P*
	*n*	Mean ± S.D.	*n*	Mean ± S.D.	
Age (years)	30	25.6 ± 3.78	20	26.5 ± 4.57	0.605
Body Mass Index (kg/m^2^)	30	26.24 ± 4.91	20	24.51 ± 3.21	**0.019**
FSH (mIU/mL)	30	5.74 ± 1.94	20	7.71 ± 2.64	**0.005**
LH (mIU/mL)	30	10.36 ± 8.68	20	4.6 ± 1.23	**0.025**
E2 (pg/mL)	30	68.89 ± 74.18	20	46.65 ± 17.8	0.452
Prolactin (ng/mL)	30	10.84 ± 5.06	20	14.46 ± 8.11	0.088
TSH (uIU/mL)	30	2.67 ± 1.51	20	2.41 ± 1.12	0.692
17-OHP (ng/mL)	30	1.59 ± 1.34	20	1.03 ± 0.43	0.088
DHEA-S (ug/dL)	30	247.2 ± 55.4	20	229.7 ± 87.6	0.428
Total testosterone (ng/mL)	30	49.48 ± 20.27	20	48.6 ± 24.24	0.707
Free testosterone (ng/mL)	30	7.20 ± 3.93	20	3.54 ± 1.18	**0.002**
HDL (mg/dL)	30	43.00 ± 8.73	20	51.25 ± 13.97	0.056
LDL (mg/dL)	30	117 ± 79.84	20	90.25 ± 24.12	0.153
OGTT (mg/dL) (2^h^glucose concentration)	30	109.6 ± 35.56	20	110.5 ± 29.2	0.451
Progesterone (pretreatment) (ng/mL)	30	3.84 ± 4.34	20	11.98 ± 5.35	**0.001**

**Table 2:** Pregnancy rates within the study and control groups with respect to glycodelin positivity.

	Pregnancy		No pregnancy		Total		*P*
	*n*	%	*n*	%	*n*	%	
Study group							
Glycodelin							
Negative	—	—	8	40.0	8	26.7	0.027
1+	4	40.0	6	30.0	10	33.3	
2+	6	60.0	6	30.0	12	40.0	
Control group							
Glycodelin							
Negative	—	—	3	37.5	3	15.0	0.030
1+	3	60.0	2	40.0	5	25.0	
2+	9	75.0	3	25.0	12	60.0	

**Table 3:** Glycodelin expression and progesterone values of patients.

	Patient number (*n*)	Progesterone (mean ± SD)	*P* value
Study group			
Glycodelin			
Negative	8	13.25 ± 10.57	0.346
Positive: 1+	10	16.9 ± 6.97	
Positive: 2+	12	20.1 ± 5.16	
Control group			
Glycodelin			
Negative	3	7.85 ± 7.10	0.542
Positive: 1+	5	12.98 ± 1.74	
Positive: 2+	12	12.59 ± 5.81	
